# Low-Level Stimulation and Ethanol Ablation of the Vein of Marshall Prevent the Vagal-Mediated AF

**DOI:** 10.3389/fcvm.2021.675485

**Published:** 2021-05-05

**Authors:** Fei Liu, Wei Sun, Yan Li, Yuanjun Sun, Xiaohong Yu, Xiaomeng Yin, Yunlong Xia

**Affiliations:** Department of Cardiology, First Affiliated Hospital of Dalian Medical University, Dalian, China

**Keywords:** vein of Marshall, vagal atrial fibrillation, ganglionated plexi, low-level stimulation, ethanol ablation

## Abstract

**Background:** The mechanisms for the vein of Marshall (VOM) mediated atrial fibrillation (AF) are not completely understood. We sought to evaluate the contribution of the intrinsic cardiac autonomic nervous system in VOM mediated AF.

**Method:** Seven mongrel dogs were administered propranolol and continuously exposed to left superior ganglionated plexi (LSGP) stimulation, LSGP + low-level VOM stimulation, LSGP + atropine administration, LSGP + VOM filling with ethanol separately. The effective refractory period (ERP) and window of vulnerability (WOV) at the left superior pulmonary vein (LSPV), left inferior pulmonary vein (LIPV) and left atrial appendage (LAA) were measured.

**Result:** LSGP stimulation significantly shortens the ERP and prolonged the ERP dispersion and WOV in LSPV, LIPV, and LAA. Interestingly, low-level VOM stimulation, atropine administration, or VOM filling with ethanol were able to attenuate the effects of LSGP in all sites.

**Conclusion:** VOM as an inter-communication pathway of ganglionated plexis plays an important role in the development of vagal-related AF.

## Introduction

The concept of vagal atrial fibrillation (AF) was first put forward by Coumel, who suggested that the autonomic nervous system plays a pathophysiological role in a subset of patients with AF (1, 2). This was further supported in experiments by Liu and Nattel, whose work demonstrated a shortening of the action potential and refractory period creating heterogeneity across the atrial wall and a substrate for re-entrant arrhythmogenesis (3). Subsequent evidence has further shown that vagal stimulation causes potent increases in the heterogeneity of the atrial effective refractory period (ERP). far over that caused by sympathetic stimulation (3). Recently, intrinsic cardiac nerves (ICNs) and their associated ganglionated plexi (GP) have emerged as potential anatomical sites for the initiation and maintenance of AF by involving the parasympathetic nervous system. Stimulation of these areas can trigger AF in humans (4, 5). Besides, high-frequency stimulation of GP at the pulmonary vein can induce ectopy and AF (1). Other studies in human subjects documented shifts in autonomic tone toward vagal predominance in the initiation of paroxysmal AF (potentially following an initial sympathetic surge), and eventually enhances the occurrence of ectopy at the site of the pulmonary veins (6).

The vein of Marshall (VOM) and ligament of Marshall (LOM) have been implicated in the pathogenesis of AF. Both VOM and LOM, sources of initiating triggers (7), and vehicles of parasympathetic (8) and sympathetic (9) innervations that modulate electrical properties of atrial tissue, contribute to AF maintenance (10, 11). Animal and human studies have shown that the VOM and LOM is a potential therapeutic target (12–14) for AF. In the past, high-frequency stimulation (HFS) in the LOM (without exciting the atrial myocardium) led to induction of AF, and this induction was inhibited by esmolol and atropine administration, suggesting the involvement of autonomic mediation (15, 16). Here, we hypothesized that VOM, as a key inter-communication pathway of GP, may play an important role in the development of vagal-related AF. Therefore, this study was carried out to reduce AF vulnerability by evaluating the effect of VOM ethanol infusion to the intrinsic cardiac autonomic nervous system.

## Methods

### Animal Model Preparations

**Figure 1:**
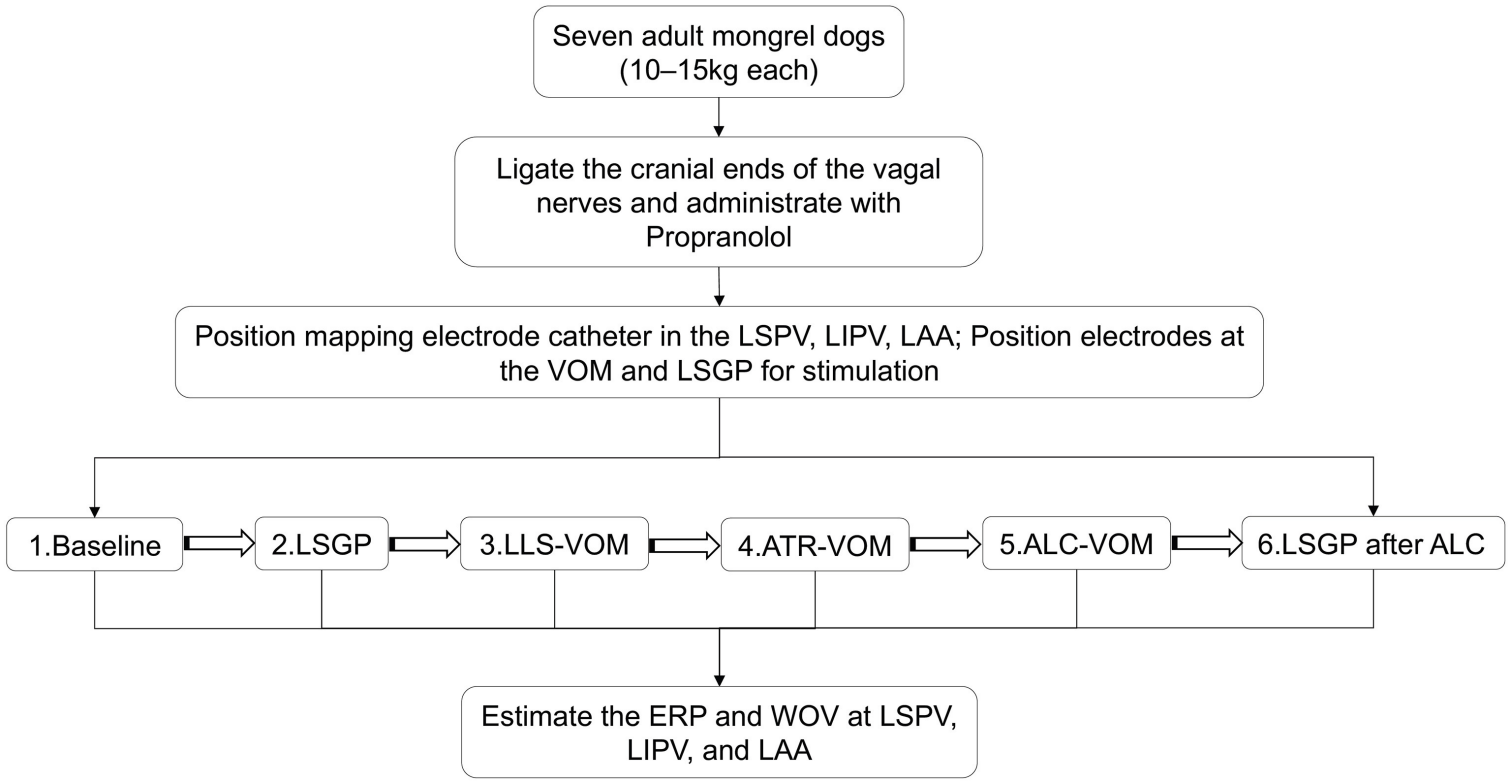
Schematic of the study design.

The Institutional Animal Care and Use Committee of Dalian Medical University approved the experimental protocol in advance. Seven adult mongrel dogs (10–15 kg each) were anesthetized with sodium pentobarbital (150 mg/kg intravenously). The dogs were ventilated with a constant volume-cycled respirator through a cuffed endotracheal tube, and blood oxygen saturation was maintained above 95%. The temperature and illumination of the operating room were kept stable throughout the experiment. Standard ECG leads II and aVR was continuously monitored (Prucka 7000; GE Healthcare, Milwaukee, WI). All dogs were administered propranolol, initially a loading dosage of 2-mg/kg bolus, and subsequently a maintenance dosage of 2 mg/kg per hour to inhibit sympathetic activity. All the mongrel dogs continuously underwent the standard procedure as represented in Figure 1.

### Catheter Positioning

**Figure 2:**
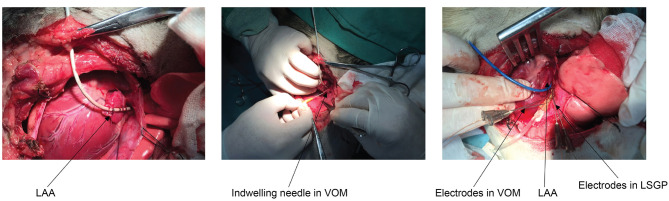
Anatomical location and catheter positioning. LAA, left atrial appendage; VOM, vein of Marshall; LSGP, left superior ganglionated plexi.

All underwent a left lateral thoracotomy at the fourth intercostal space and the pericardium was opened to expose the left atrial appendage (LAA) and the base of the left ventricle. At the lateral aspect of the LAA, the LOM as a prominent fold was clearly visualized. A mapping electrode catheter was positioned, and sutured to fix it in position at the left superior pulmonary vein (LSPV), left inferior pulmonary vein (LIPV), and LAA to promote the recording of the signals to estimate the effective refractory period (ERP) and window of vulnerability (WOV). In addition, electrodes were deployed for stimulation purposes at the VOM and left superior ganglionated plexi (LSGP). All catheters were manufactured by Cordis, Biosense Webster, Inc. (Diamond Bar, CA) as shown in Figure 2.

### LSGP Stimulation

Two pairs of electrodes were embedded in the caudal end of the LSGP for stimulation. A rectangular pulse was delivered through a constant voltage stimulator at 20 Hz with a pulse width of 2 ms by a programmable stimulator (RST-2, Huanan Medical, Hunan, China). The LSGP stimulation threshold was defined as the voltage level that could decrease the heart rate (HR) by 30% or result in a 2:1 atrioventricular block. The threshold voltage×2 was used to test the atrial ERP.

### Low-Level VOM Stimulation

We first defined the stimulation threshold for each dog by stimulating the VOM at 13 Hz (450-μs pulse duration). The stimulus amplitude (V) that elicited an abrupt decrease of heart rate by >20% from baseline or caused AV conduction block was defined as the stimulation threshold. We then programmed the pacemaker output to 1 V below the stimulation threshold and confirmed that this stimulus voltage (4 ± 2 V, range 1–6 V) did not cause any changes in heart rate. The stimulation parameters chosen resulted in no serious adverse reactions (17).

### VOM Ethanol Infusion

Selective cannulation of the VOM was reliably achieved by introducing a left internal mammary artery catheter in the coronary sinus, engaging the VOM ostium. Through the catheter, an angioplasty wire and a balloon catheter (2 mm × 8 mm) were advanced into the VOM. The balloon was inflated at the ostium of the VOM and selective VOM venograms were obtained. Substantial anatomical variations were present in the length and caliber of the VOM. The angioplasty balloon was then inflated at the ostium of the VOM and 5 cc of 100% ethanol was delivered over 2 min through the balloon lumen.

### ERP and WOV Measurement

ERP and WOV at LSPV, LIPV, and LAA were measured under baseline, LSGP stimulation alone, LSGP stimulation + low-level VOM stimulation, LSGP stimulation+ atropine administration, VOM ethanol infusion alone, and VOM ethanol infusion+ LSGP stimulation respectively. ERP was measured by an incremental technique, with 2-ms steps at basic drive cycle lengths of 500 ms for eight beats. ERP was measured with a drive train of eight stimuli (S1 = 400 ms) at twice the diastolic threshold, followed by a single premature stimulus (S2) coupled with increments of 10 ms until local ERP was reached. Atrial ERP was defined as the longest S_1_-S_2_ coupling interval that failed to produce a propagated response. We used the WOV as a quantitative measure of AF inducibility. During ERP measurements, if AF was induced by decremental S1-S2 stimulation, the longest and the shortest S1-S2 interval (in ms) at which AF was induced were then determined. The difference between the two was designated as the WOV. AF was defined as ≥2 s of atrial activity appearing as fibrillatory waves on the surface ECG. Whereas, ERP dispersion was defined as the coefficient of variation (standard deviation/ mean) of the ERP at LSPV, LIPV, and LAA Sites.

### Statistical Analysis

A paired *t*-test was used for comparisons of ERP and WOV before and after stimulations. ANOVA for repeated measurements was used for comparisons of ERP or WOV among different stimulation strategies and followed by *post hoc* testing (least significant differences) for comparisons of the ERP and WOV at the end of each subsequent stimulation strategy vs. ERP and WOV in the baseline state. Statistical significance was defined as *P* ≤ 0.05.

## Results

### Effect of LSGP Stimulation Mimics Vagal Stimulation

**Figure 3:**
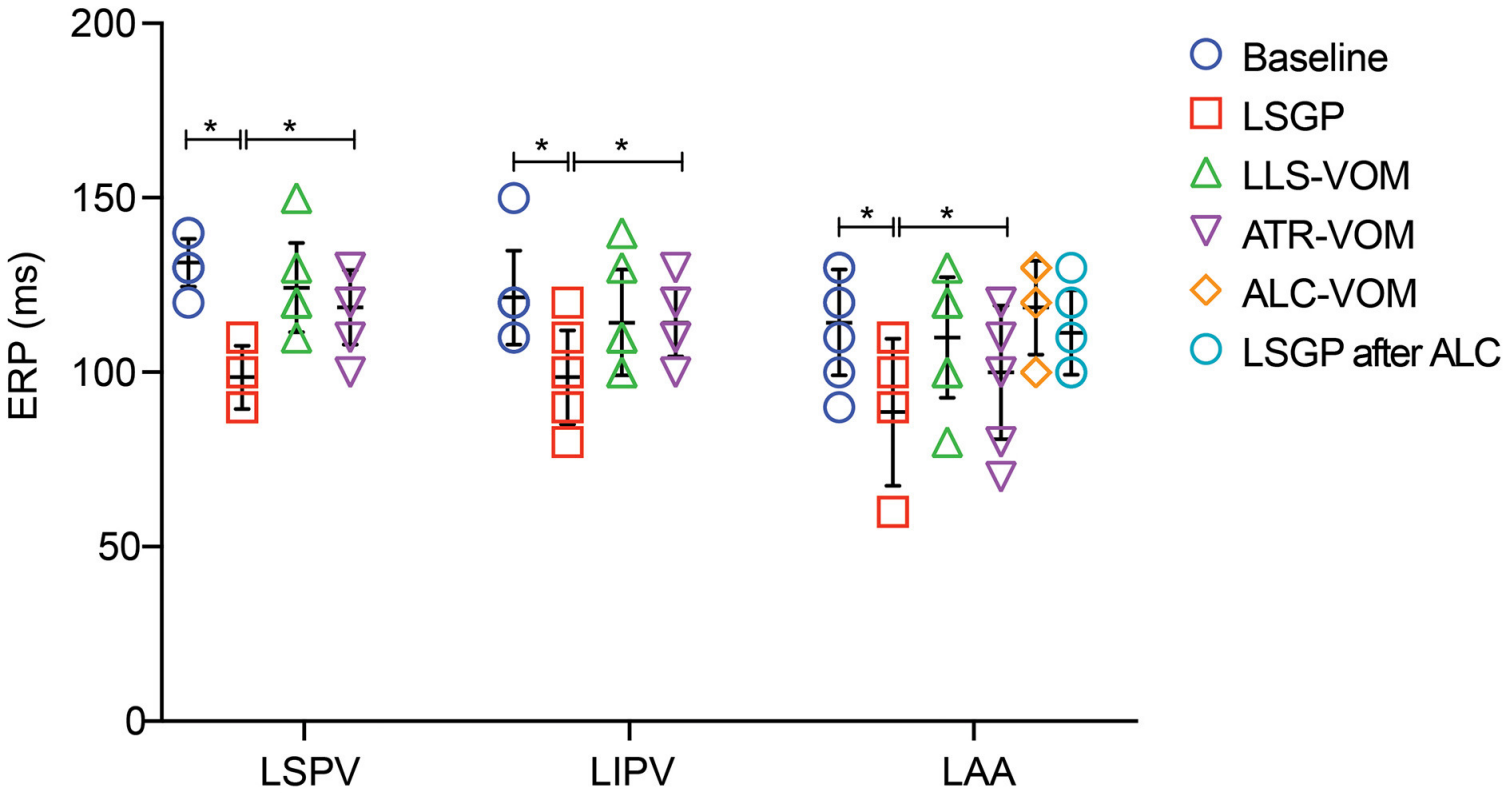
Effective refractory period at left superior pulmonary vein (LSPV), left inferior pulmonary vein (LIPV), and left atrial appendage (LAA). LSGP, left superior ganglionated plexi; LLS, low-level VOM stimulation; ATR, atropine; ALC, alcohol; VOM, vein of Marshall; *P < 0.05.

**Figure 4:**
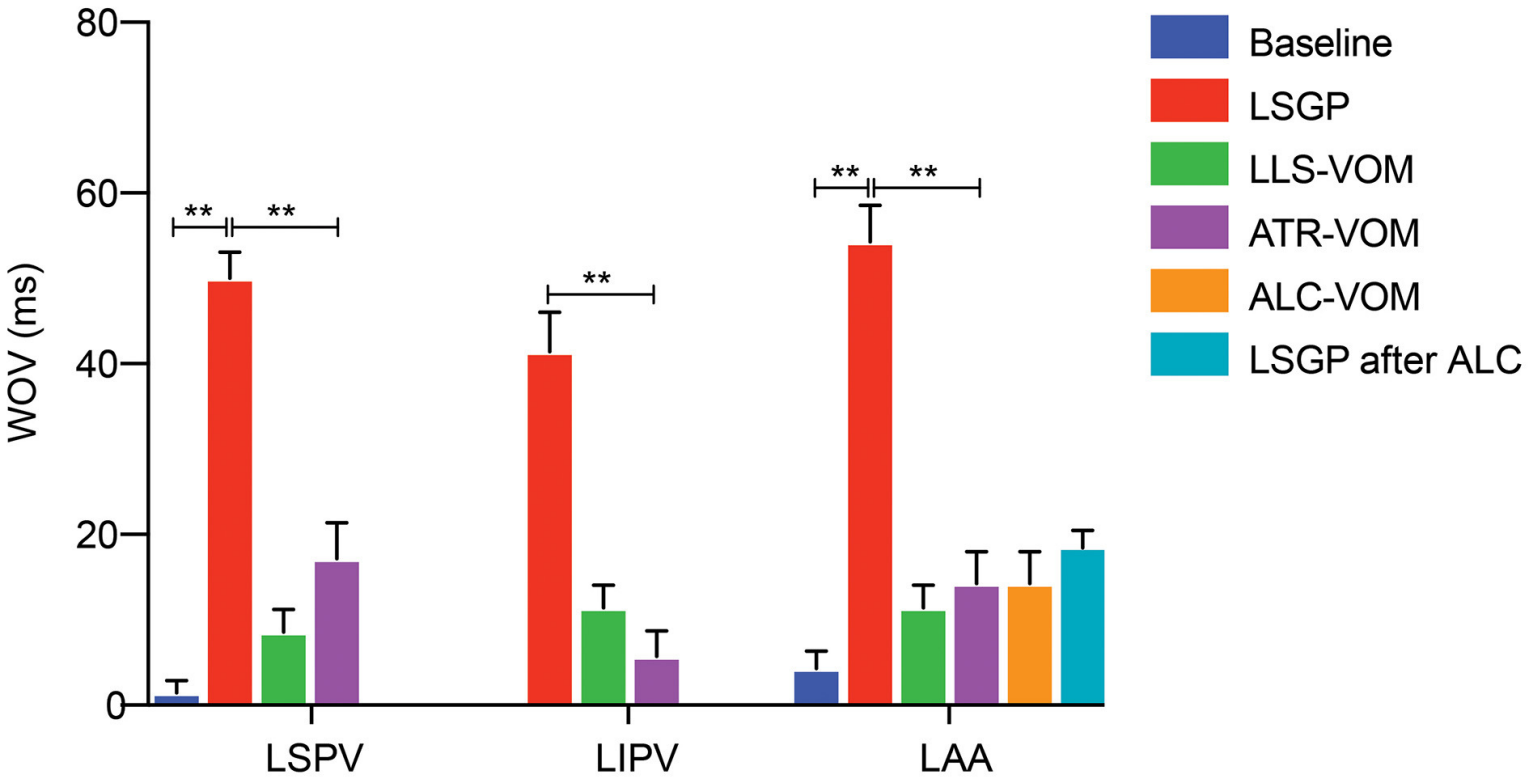
WOV at LSPV LIPV and LAA; **P < 0.01.

As shown in Figure 3, LSGP stimulation markedly decreased the ERP in LSPV (98.6±9.0 ms vs. 131.43 ± 6.9ms, *p* < 0.001), LIPV (98.6 ± 13.5 ms vs. 121.4 ± 13.5 ms, *p* = 0.016), and LAA (88.6 ± 21.2 ms vs. 114.3 ± 15.1 ms, *p* = 0.067). Meanwhile, the ERP dispersion and WOV were increased significantly after LSGP stimulation (Figure 4). The ERP dispersion increased from 0.10 ± 0.04 to 0.16 ± 0.08 with a *P*-value of 0.032. Also, the WOV at LSPV, LIPV, and LAA was significantly prolonged after LSGP stimulation from 1.4±3.8 ms, 0 ms, to 4.3 ± 5.4 ms in the baseline state to 50 ± 8.2 ms, 41.4 ± 12.2 ms, and 54.3 ± 11.3 ms, respectively.

### LSGP + Low-Level VOM Stimulation Attenuates the Effects of LSGP

**Figure 5:**
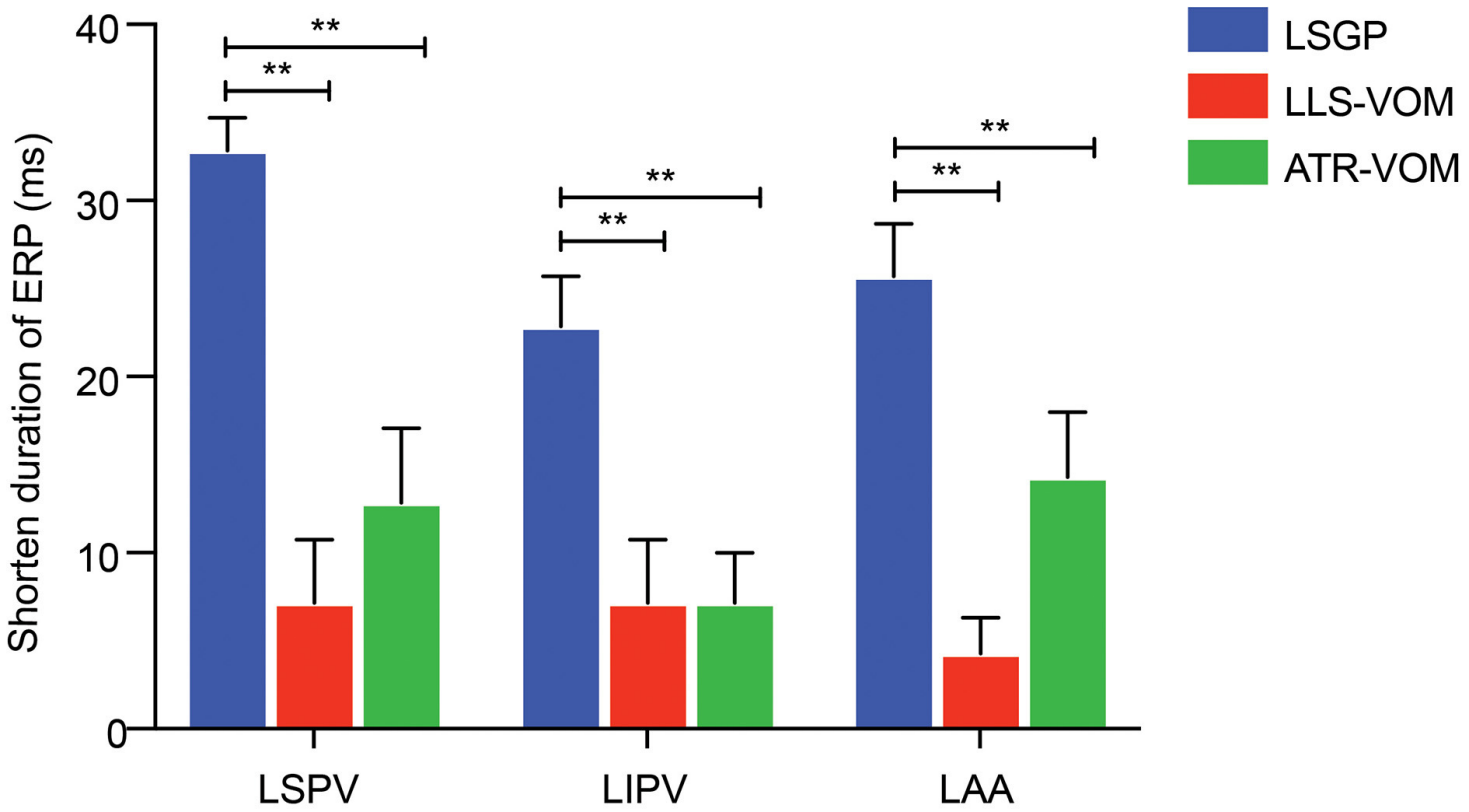
Shorten duration of ERP at LSPV, LIPV, and LAA; **P < 0.01.

Compared to the LSGP right before VOM stimulation, the shorten duration of ERP was less in LSPV (98.6 ± 9.0ms vs.124.3 ± 12.7ms, *p* < 0.001), LIPV (98.6 ± 13.5 ms vs. 114.3 ± 15.1 ms, *p* = 0.140), and LAA (88.6 ± 21.2 ms vs. 110.0 ± 17.3 ms, *p* = 0.183), when LSGP combined with low-level VOM stimulation (Figure 5). Similarly, the ERP dispersion was hardly increased (0.13 ± 0.03). However, the WOV at LSPV (8.6 ± 6.90 ms), LIPV (11.4 ± 6.90 ms), and LAA (11.4 ± 6.90) were less prolonged with low-level stimulation in VOM.

### LSGP + Atropine Administration Attenuates the Effects of LSGP

In comparison with the ERP measured from LSGP alone, the addition of atropine to LSGP slightly shorten the ERP from 131.4 ± 6.90 ms, 121.4 ± 13.5 ms, and 114.3 ± 15.2 ms to 118.6 ± 10.7 ms, 114.3 ± 9.8 ms, and 100.0 ± 19.2 ms in LSPV, LIPV, and LAA respectively. However, there were no significant differences in ERP. The growth rate in ERP dispersion (0.12 ± 0.06) and WOV was significantly reduced when atropine administration was added to LSGP stimulation.

### LSGP + VOM Filling With Alcohol Attenuates the Effects of LSGP

There was no significant difference in baseline ERP in LAA before (114.3 ± 15.1 ms) and after VOM filling. Likewise, LSGP fails to significantly shorten the ERP in LAA (107.1 ± 18.6 ms) when VOM was filled with alcohol. Also, the baseline WOV was not significantly changed after VOM filling in LSPV (1.4 ± 3.8 vs. 0 ms) and LIPV (0 vs. 0 ms). However, the WOV slightly increased in LAA after VOM filling (14.3 ± 9.8 vs. 4.3 ± 5.4ms). LSGP stimulation was also unable to significantly prolong the VOM in LSPV (0 vs. 0 ms), LIPV (0 vs. 0 ms), and LAA (14.3 ± 9.8 vs. 18.6 ± 7.0).

## Discussion

The present study demonstrated that low-level stimulation, atropine mediation, or VOM filling with alcohol could attenuate the GP-activation-induced shortening of atrial ERP and the increase of AF inducibility, suggesting VOM is being involved in the development of vagal-related AF as an inter-communication pathway of GPs.

The intrinsic cardiac nervous system harbors clusters of neurons, the GP, which innervate the neighboring atrial myocardium and control their electrophysiological properties (18). As shown in our study, LSGP stimulation markedly decreased the ERP while significantly increased the ERP dispersion and WOV, which is consistent with previous studies. The result of our study demonstrates that cardiac autonomic nerves and GPs play an important role in the initiation and maintenance mechanisms of AF (19). GP, as a target during AF ablation, has been reported to be useful to improve the outcomes (20). However, the benefit of GP ablation is still controversial. There are complex interneural communications between different ICN (16), and GP ablation that could damage various nerve structures, myocardium, and other non-neuronal structures is not highly recommended as it leads to proarrhythmia in return (21).

The VOM, coming from the ligament of Marshall running between the left atrial appendage and left pulmonary vein antrum, is an intracardiac structure connects with the coronary sinus. Several studies showed the nervous system forms an interconnected neural network composed of GPs and VOM (15, 22). Valderrabano demonstrated that the VOM contains ICN that connects with the AV node and can trigger AF and retrograde ethanol infusion in the VOM reliably eliminates local ICN responses (14). Lin indicated that the VOM and ILGP function as the “integration centers” that modulate the autonomic interactions between extrinsic and intrinsic cardiac ANS on AV nodal function (16). ICN reached by VOM ethanol infusion may include not only those ICN clustered in the VOM-posterolateral left atrial ganglionated plexus but also those associated with the inferior left ganglionated plexus (23). In our study, we found that the LSGP stimulation result in short ERP and long ERP dispersion and WOV. Importantly, the changes were alleviated when VOM was filled with alcohol. These phenomena confirm that the VOM is an important inter-communication pathway of GPs, and its participation in the development of vagal-related AF.

The vagus nerve has been proved to exert inhibitory control over the autonomic ganglia and autonomic neuromodulation. A shred of evidence reported that low-level vagus nerve stimulation could inhibit the activity of the autonomic ganglia and reverses acute electrical atrial remodeling during rapid atrial pacing (17, 24, 25). Similarly, our study found low-level stimulation of VOM markedly attenuates the effect of LSGP, which may also contribute to autonomic inhibition. Although the exact downstream targets of LLVNS remain to be determined, there is preliminary evidence that the involvement of the antiadrenergic neuropeptide vasostatin-1 (26), the nitric oxide signaling pathway (27), and up-regulation of the calcium-activated potassium channel 2 in the left stellate ganglion (28). Thus, the potential mechanism for the low-level stimulation of VOM as a treatment for AF needs to be further investigated.

## Limitation

There are several limitations to the present study. Firstly, the small sample size; secondly, the study was carried out only in animal model thus data on cell line and human is limited. Thirdly, sequential application of different interventions may result in carry over effects, which may limit interpretation of the results.

## Conclusion

VOM as an inter-communication pathway of GPs is involved in the development of vagal-related AF. Mediation with either low-level stimulation or atropine administration could attenuate the GP-activation-induced shortening of atrial ERP and the increase of AF inducibility. Destroyed VOM with alcohol further confirmed the critical role of VOM.

## Data Availability Statement

The raw data supporting the conclusions of this article will be made available by the authors, without undue reservation.

## Ethics Statement

The animal study was reviewed and approved by Ethics Committee of First Affiliated Hospital of Dalian Medical University.

## Author Contributions

XYi and YX designed this study. YS and XYu were in charge of data analysis and critical revision of the article. FL and WS drafted the article. FL, WS, and YL conducted the animal experiments and data collection. All authors have read and approved the final manuscript.

## Conflict of Interest

The authors declare that the research was conducted in the absence of any commercial or financial relationships that could be construed as a potential conflict of interest.

## Abbreviations

AF: atrial fibrillation
ERP: effective refractory period
GP: ganglionated plexi
ICN: intrinsic cardiac nerves
VOM: vein of Marshall
LOM: ligament of Marshall
HFS: High-frequency stimulation
LSPV: left superior pulmonary vein
LIPV: left inferior pulmonary vein
LAA: left atrial appendage
LSGP: left superior ganglionated plexi
LLS: low-level VOM stimulation
ATR: atropine
ALC: alcohol.
